# *Confianza*, culture, and care: barriers to and facilitators of mental health treatment engagement among Latinos with serious mental illness

**DOI:** 10.3389/fpsyg.2026.1904976

**Published:** 2026-08-21

**Authors:** Oscar F. Rojas Perez, Monique T. Cano, Andrea Mendiola, Luis M. Añez Nava, Marilyn Perez Avilés, Estela Meléndez, Manuel Paris, Michelle A. Silva

**Affiliations:** 1 Department of Psychiatry, Yale University School of Medicine, New Haven, CT, United States; 2 NORC, University of Chicago, Chicago, IL, United States; 3 Advocates for Basic Legal Equality, Inc., Toledo, OH, United States

**Keywords:** barriers, CFIR, engagement, facilitators, Latinos, serious mental illness

## Abstract

**Introduction:**

Among the 3.2 million Latinos in the United States with serious mental illness (SMI) in 2024, less than 15.5% received any treatment or medication. Research on mental health treatment engagement among Latinos has largely conceptualized engagement broadly, overlooking nuances in the engagement process, disorder severity, and community heterogeneity. Most studies on treatment engagement among Latinos with SMI have explored macro-level factors without a guiding framework or theory. Thus, the aim of the current study was to identify barriers to and facilitators of treatment engagement among Latinos with SMI using the Consolidated Framework for Implementation Research (CFIR).

**Methods:**

Sixteen Latino adults with SMI (*n* = 8 in treatment; *n* = 8 not in treatment) participated in qualitative semi-structured interviews in their preferred language via Zoom. The interview questions were informed by the CFIR and the available literature on Latinos with serious mental illness. The interview questions were reviewed and revised through a collaborative validation process with an all-Latino community advisory team.

**Results:**

Thematic analysis of the data identified a range of barriers to and facilitators of engagement in mental health treatment. Inner setting domain barriers included incongruence between treatment and Latino culture, and limited availability of bilingual and bicultural providers. Individual characteristic domain barriers included negative experience with mental health providers, lack of trust in providers and systems of care, and mental health stigma. Inner setting domain facilitators included mental health psychoeducation and providers who listen and are compassionate. Individual characteristics domain facilitators included preferences for behavior-based, context-based, and faith-based care.

**Conclusion:**

These findings contribute to existing literature by shedding light on non-social determinants of health (SDOH)-related barriers to and facilitators of treatment engagement for Latinos with SMI.

## 1 Introduction

Serious mental illness (SMI), disorders that substantially interfere with an individual’s life and ability to function, are a significant cause of morbidity and mortality across ethno-racial groups (Breslau et al., 2005; Chang et al., 2010; Corrigan et al., 2017; Parks et al., 2006; Substance Abuse and Mental Health Service Administration, 2024). These disorders (e.g., major depressive disorder, bipolar disorder, schizophrenia) are typically left untreated, particularly among Latinos in the United States (Substance Abuse and Mental Health Service Administration, 2024). Among the 3.2 million Latinos in the United States with SMI in 2024, less than 15.5% received any treatment or medication (Substance Abuse and Mental Health Service Administration, 2024). These outcomes disproportionately affect Latinos with limited English proficiency, many of whom are underinsured, highly vulnerable, and experience longer periods of untreated disorders (Falgas et al., 2017; Gonzalez et al., 2011; Maura and Weisman de Mamani, 2017). The evidence also suggests that Latinos with SMI not engaged in mental health treatment experience inequities in service utilization (Green et al., 2020). These disparities are compounded by structural and attitudinal barriers, including stigma, all of which contribute to poor psychiatric outcomes among Latinos with SMI (Green et al., 2020; Cabassa et al., 2007; Interian et al., 2007; Vega et al., 2010). Consequently, understanding factors that influence treatment engagement among Latinos with SMI is critical to reducing disparities in mental health care.

Research on mental health treatment engagement among Latinos has largely conceptualized engagement broadly, overlooking nuances in the engagement process, disorder severity, and community heterogeneity. This broad approach has provided fundamental knowledge on structural and cultural barriers to mental health treatment engagement for common mental disorders among Latinos (Green et al., 2020; Caplan and Whittemore, 2013; Chavira et al., 2014; Uebelacker et al., 2012; Wells et al., 2013). However, fewer studies have examined whether these broadly identified barriers operate similarly among Latinos with SMI or across different dimensions of engagement (Evans et al., 2016; Gilmer et al., 2007). Even less is known about the facilitators of treatment engagement for Latinos with SMI (Corrigan et al., 2017; Corrigan et al., 2018). Together, these limitations highlight the need for more nuanced research on treatment engagement among Latinos with SMI.

Another limitation of the existing literature on treatment engagement among Latinos with SMI is the lack of studies grounded in conceptual frameworks (Uebelacker et al., 2012; Cabassa et al., 2008). Most studies on treatment engagement among Latinos with SMI have explored macro-level factors without a guiding framework or theory (Caplan and Whittemore, 2013; Barrio et al., 2008; Martínez Pincay and Guarnaccia, 2007). Exploring treatment engagement factors without a guiding framework or theory may limit knowledge of the complex and multilevel factors influencing treatment engagement among Latinos with SMI. Thus, the aim of the current study was to identify barriers to and facilitators of treatment engagement among Latinos with SMI using the Consolidated Framework for Implementation Research [(CFIR Damschroder et al., 2009)]. Applying the CFIR to treatment engagement will broaden the field’s focus from identifying barriers alone to understanding both challenges and facilitators that can inform solutions. To the author’s knowledge, the current study is among the first to utilize the CFIR to explore barriers to and facilitators of treatment engagement among Latinos with SMI. The initial results are an important step toward developing culturally responsive interventions for a community that is unlikely to seek or engage in mental health treatment.

## 2 Methods

Our qualitative study was informed by the CFIR to identify barriers to and facilitators of mental health treatment engagement for Latinos with SMI (Damschroder et al., 2009; Breimaier et al., 2015; Damschroder et al., 2022). The CFIR is a comprehensive implementation science framework comprising five domains, each with a set of constructs (39 in total), intended to guide the effective implementation of evidence-based practices (Damschroder et al., 2009; Safaeinili et al., 2020). The five domains include: (1) Intervention characteristic, which explores the features and quality of the proposed intervention, and how the intervention is perceived by those responsible for its implementation; (2) Outer setting refers to the context(s) where the organization resides; (3) Inner setting alludes to structural and cultural context(s) where the implementation will take place (e.g., department, group of people); (4) Individuals pertains to attitudes, values, or beliefs of individuals regarding the intervention; and (5) Implementation process relates to the steps that promote effective implementation. The CFIR is among the most widely used frameworks in healthcare settings for predicting or explaining barriers to and facilitators of successful intervention implementation. We chose to use the CFIR for its multifaceted nature, which enabled our team to deliberately select constructs relevant to the present study’s context. The present study was also positioned within a postpositivist paradigm, which assumes the existence of an objective reality that can be interpreted from different perspectives (Morrow, 2005; Ponterotto, 2005). The authors also followed the guidelines for qualitative research reporting found in the Journal Article Reporting Standards for Qualitative Research in Psychology (Levitt et al., 2018).

### 2.1 Recruitment

Participants currently engaged in mental health treatment were recruited via clinician referral from an outpatient community mental health clinic in the northeastern United States. The clinic is well known in the community for its linguistic and culturally responsive services to monolingual Spanish-speaking Latinos with serious mental illness and/or substance use disorders (Paris et al., 2016). Participants not engaged in formal mental health treatment were recruited using English- and Spanish-language flyers posted at a Latino-serving organization within a five-mile radius of the community mental health clinic. The organization is known for providing support social services (e.g., housing, employment, education) to Latinos with SMI. All study procedures were approved by the Yale University Institutional Review Board (Human Investigation Committee #2000034687).

### 2.2 Participant eligibility and screening

The lead author administered a brief structured screener to all individuals interested in the study. Eligibility criteria included: (1) age >18 years, (2) ethnically self-identified as Latino, (3) ability to complete the study procedures in Spanish or English, (4) meet criteria for a serious mental illness based on the Substance Abuse and Mental health Services Administration definition (Substance Abuse and Mental Health Service Administration, 2024) or screened positive for a SMI via the Kessler Psychological Distress Scale (K6) (Ferro, 2019), (5) ability to provide informed consent, and (6) currently engaged or not engaged in formal mental health treatment.

During screening, participants were asked whether they had ever received a formal diagnosis of SMI from a provider and about their treatment history. The lead author asked each participant to identify their formal primary diagnosis (e.g., major depressive disorder, bipolar disorder, or schizophrenia). For participants in treatment, formal diagnoses were confirmed by the referring provider. Participants not engaged in formal mental health treatment also completed the K6. Designed to screen for severe mental illness in the general population, K6 scores >13 were considered indicative of the diagnostic threshold for a severe mental illness (Prochaska et al., 2012). Table 1 displays participants’ mental health diagnoses, physical health conditions, and medication adherence.

**Table 1:** Participant diagnoses by treatment status.

Mental/physical conditions and medication status	Treatment *n* (%)	Not in treatment *n* (%)
What is your long term diagnosed mental health condition?	*N* = 8	*n* = 8
Schizophrenia	2 (25.0)	–
Bipolar disorder	3 (37.5)	3 (38.0)
Major depressive disorder with psychotic features	3 (37.5)	–
Major depressive disorder	–	5 (62.0)
What other long term mental health conditions have you been diagnosed with?	*N* = 7	*n* = 4
Generalized anxiety disorder	4 (57.1)	1 (24.0)
Major depressive disorder	1 (14.3)	3 (75.0)
Panic disorder	1 (14.3)	–
Bipolar disorder	1 (14.3)	–
What are your long term diagnosed physical health conditions?	*N* = 2	*n* = 2
Diabetes	–	2 (100)
OCPD	1 (50.0)	–
Asthma	1 (50.0)	–
Medication for mental health disorder		
Antipsychotic (yes)	8 (100)	–
Antipsychotic (no)	–	8 (100)

Participants were asked to write in all formal mental and physical health disorders diagnosed by a Psychologist, Psychiatrist, or Doctor of Medicine, and medication prescribed.

### 2.3 Interviews

Participants who expressed interest in the study contacted the lead author. Next, participants were screened to determine eligibility and given additional details about the study, including confidentiality, the option to withdraw from participation, and compensation (a Walmart or Target gift card valued at $40 upon completion). Eligible participants were given the option to complete the interview in person, virtually via a Health Insurance Portability and Accountability Act (HIPAA)-compliant version of the Zoom videoconferencing platform (Zoom Communications, 2025), or via phone with the lead author. Participants then completed a secure consent form, followed by a demographic questionnaire. The interviews were conducted from January to August 2024, each lasting 60–90 min and in the participants’ preferred language. All 16 interviews were conducted via Zoom by the lead author.

### 2.4 Interview guide

The interview questions were informed by the CFIR and the available literature on Latinos with serious mental illness (Damschroder et al., 2009; Patton, 2014). Subsequently, the interview questions were reviewed and revised through a collaborative validation process with an all-Latino community advisory team composed of two psychologists, a psychiatrist, two peer recovery support specialists, a language coordinator for immigration legal aid, and a doctoral-level psychology trainee (Urry et al., 2024). The final interview questions explored topics such as lived experience with a serious mental illness and service utilization, barriers to and facilitators of mental health services, treatment congruency with Latino culture, and cultural methods of healing mental health conditions. Supplementary Appendix 1 displays the interview protocol.

### 2.5 Interviewer background

The semi-structured interviews were conducted by the lead author, a licensed bilingual and bicultural psychologist of Guatemalan ethnic background. At the time, he held a faculty appointment in the psychology section within the department of psychiatry at a medical school in the northeastern United States. His research broadly focuses on Latino mental health, cultural adaptation of mental health interventions, and alcohol use among priority populations. The lead author has extensive knowledge and experience conducting qualitative studies (Cano et al., 2025; Castellanos et al., 2020; Rojas Perez et al., 2022).

### 2.6 Reflexivity statement

The lead author’s personal and professional background shaped all stages of the research project, including study design and data analysis. The coding team included the lead author (OFRP), a second faculty member (MTC), and a fourth-year undergraduate student (SM), all of whom identify as bilingual and bicultural. Coder MTC is a licensed clinical psychologist of Mexican ethnic background and, at the time, held a faculty appointment in the department of psychiatry at a medical school in the northeast. Coder SM is a fourth-year undergraduate student of Peruvian ethnicity, pursuing a bachelor’s degree in medical anthropology at a small northeastern university.

The two faculty members have extensive experience conducting mixed-methods research and providing clinical care to Latinos with varying mental health disorders and substance use disorders. SM has extensive knowledge of how political-economic factors shape the mental health of Latinos and healthcare systems. Thus, the coding team’s diverse representation offered a critical perspective for examining the data. Before and throughout the analysis, the coding team reflected on how their personal and professional backgrounds influenced their perspectives on the study topic and approach to data exploration. Throughout the analytic process, the coding team engaged in reflective discussions on how being mental health scientist-practitioners can introduce biases in data interpretation. As a result, there is a risk of emphasizing professional knowledge at the expense of fully centering participants’ lived experience. The ongoing reflective and open dialogue helped the coding team mitigate the likelihood of overlooking or undervaluing participants’ lived experiences, regardless of shared or differing backgrounds.

## 3 Data analysis

A HIPAA-compliant transcript service company transcribed the audio-recorded interviews verbatim. Transcripts were checked for accuracy by the lead author. To build trustworthiness, our coding team employed analytic and investigator triangulation by considering multiple interpretations, using multiple coders, and consulting with an all-Latino community panel of experts (Patton, 2014; Carter et al., 2014; Elo and Kyngäs, 2008).

Data were analyzed using Dedoose Qualitative Research Software version 10.0.59 (Socio Cultural Research Consultants, 2026). Dedoose is a secure cloud-based application designed for real-time collaborative analysis of qualitative data. The software organizes coding using a hierarchical, tree-structured codebook, allowing coders to create parent codes and subcodes. Interview transcripts were analyzed using Braun and Clarke (2019) and Braun and Clarke (2022) reflexive thematic analysis, given its flexibility, and in their original language (Spanish or English). A deductive and inductive coding approach was implemented to explain barriers to and facilitators of engagement in formal mental health treatment among Latino adults with serious mental illness. Our coding team followed the six sequential steps outlined by Braun and Clarke (2006). In the first step, data familiarization, each member of the coding team independently read the transcripts and documented initial ideas and codes. In the second step, a list of initial codes was generated and discussed. The coding team then coded relevant information that reflected the study’s objective. During weekly meetings, the coding team reviewed and compared coded transcripts to identify commonalities and inconsistencies. The coding team resolved discrepancies through in-depth discussion until consensus was established. Each transcript was independently double-coded and reviewed by the coding team. In the third step, initial themes were generated from the codes, and relevant data were organized for each theme. In the fourth step, the coding team reviewed and discussed the initial theme until consensus was achieved. In the fifth step, the themes were named and further consolidated into the Inner setting and Individual characteristics domains of the CFIR. The lead author then presented and discussed themes with the all-Latino community panel of experts. In the final step, the coding team identified and discussed excerpts that clearly reflected the themes. Excerpts in Spanish were translated into English by the lead author and back-translated into Spanish by the second author. Finally, themes were established based on reporting patterns observed among most participants.

## 4 Results

### 4.1 Sample characteristics

A total of *N* = 16 participants aged 19–73 years (*M* = 47.69, *SD* = 16.08), with *n* = 9 (56%) identifying as women, *n* = 10 (62%) Puerto Rican, and *n* = 12 (75%) U. S. citizens, completed the study. The majority reported an annual income of less than $25,000; *n* = 13 (81%) were primarily Spanish-speaking, *n* = 7 (44%) were single, and *n* = 13 (81%) were Catholic. Half of the participants reported seeking services at federally qualified health centers. Table 2 displays participants’ characteristics.

**Table 2:** Demographics by treatment status.

Variable	Treatment			Not in treatment		
	M (SD)	*n*	%	M (SD)	*n*	%
Age, in years	50.13 (11.75)			45.25 (20.00)		
Gender identity						
Female		5	62.5		4	50.0
Male		2	25.0		4	50.0
Transgender		1	12.5		–	–
Ethnic country of origin						
Cuba		1	12.5		–	–
Guatemala		1	12.5		–	–
Mexico		2	25.0		–	–
Puerto Rico		3	37.5		7	87.5
El Salvador		1	12.5		–	–
Ecuador		–	–		1	12.5
Documentation status						
Undocumented		3	37.5		1	12.5
U. S born citizenship		4	50.0		7	87.5
Naturalization citizenship		1	12.5		–	–
Income level						
No income		–	–		2	25.0
Less than $25,000		8	100		3	37.5
$25,000–$50,000		–	–		1	12.5
$50,000–$100,000		–	–		2	25.0
Primary language						
Spanish		7	87.5		6	75.0
English		1	12.5		2	25.0
Relationship status						
Civil union		1	12.5		2	25.0
Married		1	12.5		2	25.0
Divorced		1	12.5		2	25.0
Single		5	62.5		2	25.0
Religious affiliation						
Agnostic		1	12.5		–	–
Christian		6	75.0		7	87.5
Spiritual, but not religious		1	12.5		1	12.5
Service utilization center						
Federally qualified community health center		7	87.5		1	12.5
Private practice		1	12.5		–	–
Hospital		–	–		3	37.5
No place		–	–		4	50.0

Specialist degree included Ed. S. or Sp. Ed. Service utilization for participants not in treatment refers to past mental health service use.

#### 4.1.1 Qualitative findings

In our analysis, participants identified a range of barriers to and facilitators of mental health treatment engagement. Table 3 summarizes the barriers and facilitators that influence treatment engagement based on the Inner setting and Individual characteristics of the CFIR domains. Inner setting domain barriers included incongruence between treatment and Latino culture, and limited availability of bilingual and bicultural providers. Individual characteristic domain barriers included negative experience with mental health providers, lack of trust in providers and systems of care, and mental health stigma. Inner setting domain facilitators included mental health psychoeducation and providers who listen and are compassionate. Finally, the Individual characteristics domain facilitators included preferences for behavior-based, context-based, and faith-based care.

**Table 3:** Barriers to and facilitators of treatment engagement.

CFIR domain	CFIR construct	Theme(S)	Treatment	Not in treatment
Barriers				
Inner setting				
	Tension for change	Incongruence between treatment and Latino culture	*n* = 1	*n* = 7
	Available resources	Limited availability of Spanish-speaking culturally responsive providers	*n* = 4	*n* = 8
Individual characteristics				
	Need	Negative experience with mental health providers	*n* = 8	*n* = 5
	Need	Lack of trust in providers and systems of care	*n* = 6	*n* = 6
	Need	Mental health stigma	*n* = 7	*n* = 8
Facilitators				
Inner setting				
	Access to knowledge and information	Mental health psychoeducation	*n* = 4	*n* = 5
	Relational connection	Providers who listen and are compassionate	*n* = 8	*n* = 8
Individual characteristics				
	Need	Preference for behavioral-based interventions	*n* = 4	*n* = 4
	Need	Preference for faith-based care	*n* = 8	*n* = 8
	Need	Preference for context-based care	*n* = 4	*n* = 7

#### 4.1.2 Inner setting barriers

##### 4.1.2.1 Incongruence between treatment and Latino culture

Incongruence between treatment and Latino culture was a significant barrier discussed among participants (*n =* 1 in treatment, *n =* 7 not in treatment). The incongruence was often shaped by conflicting messages from family about treatment and from providers implementing Eurocentric interventions that opposed cultural collectivism. Many participants described providers’ misunderstandings of cultural customs, values, and norms as major obstacles to treatment engagement. One participant described how provider recommendations may invalidate or challenge familial cultural norms: “*A lot of times, I hear from therapists, “Make sure that when your parents do this, you handle it this way.” And culturally, that does not always work. I cannot be like, ‘Mom, you are invading my personal boundaries. I need you to respect them.’ That’s not going to fly in a Mexican house*.” (Woman; Age 37; In treatment). The participant’s excerpt underscores the incongruence between mainstream therapeutic approaches and the cultural values of *respeto* (respect) and *familismo* (familism) adhered to by some Latinos.

Another participant emphasized how Latino cultural expressions of warmth and *personalismo* (friendliness) may conflict with mainstream therapeutic practices that prioritize professional distancing and rigid boundaries: “*If someone is a little bit too warm or too caring, and therapists have not been exposed to that, they might see it in a way that is not positive or mistakenly think that it’s flirty*.” (Woman; Age 44; Not in treatment). The participant’s statement highlights how differences in patients’ values and therapeutic practices may hinder rapport-building, thereby negatively impacting the therapeutic alliance. For another participant, the barrier stemmed from the distrust of therapeutic confidentiality: “*These therapists could wake up one day and really tell your business to others. They really can. That takes a lot of integrity to keep your word and to follow all the HIPAA laws. But they are still human and could get mad one day and share all your information*.” (Woman; Age 25; Not in treatment). The participant’s excerpt underscores the importance of *confianza* (trust) within the therapeutic relationship, which can enhance or discourage treatment engagement.

##### 4.1.2.2 Limited availability of Spanish-speaking culturally responsive providers

Participants (*n =* 4 in treatment, *n =* 8 not in treatment) across groups described the limited availability of Spanish-speaking and culturally responsive providers as a core component of treatment accessibility, especially for participants not in treatment. Multiple participants explained the difficulties they encountered when searching for or requesting a Spanish-speaking provider. One participant expressed frustration over their struggle to find a Spanish-speaking provider and dissatisfaction with available Spanish interpreter services: “*Hay muchas clínicas que no tienen ni una persona que hable español y te ponen a hablar por teléfono con un intérprete que a veces ni siquiera sabe hablar bien el español*.” [*There are many clinics that do not have a single person who speaks Spanish, and so they put you on the phone with an interpreter who does not speak Spanish well*.] (Man; Age 58; In treatment). While interpreter services have been identified as a solution to the scarcity of bilingual providers, the participant’s excerpt highlights how quality responsive care in Spanish is unavailable. Thus, reinforcing potential negative thoughts and beliefs surrounding mental health treatment services. Meanwhile, many participants also emphasized the limited availability of bicultural providers. Participants described not only a limited number of Spanish-speaking providers, but also fewer culturally responsive providers. One participant expressed a strong preference and need for providers immersed in Latino culture to avoid having to explain their background: “*It would be nice to see more Hispanic therapists out there. It would save me a lot of time having to explain my culture to somebody who does not understand it*.” (Woman; Age 37; In treatment). The participant’s excerpt emphasizes the importance of patient and provider cultural matching, which can enhance the working alliance and strengthen *confianza*. Taken together, these quotes illustrate the need for a bilingual and bicultural workforce that is responsive to the browning of America and the nuances of Latino culture.

#### 4.1.3 Individual characteristics barriers

##### 4.1.3.1 Negative experience with mental health providers

Participants (*n =* 8 in treatment, *n =* 5 not in treatment) in both groups cited negative experiences with mental health providers as a major barrier to treatment engagement. These experiences were commonly characterized by inadequate provider follow-through, dismissal of patients’ lived experience, and a perceived lack of therapeutic benefit. One participant described how prior negative experiences with a provider that implemented a question-answer therapeutic approach discouraged them from seeking help as an adult: “*When I went to therapy, keep in mind I was 13, I would just sit down and answer questions, and that’s pretty much it. And once I realized that, I was like, ‘dang, this is not helping me in any way.’ So, there is no benefit to treatment now*.” (Man; Age 19; Not in treatment). The participant’s statement reflects how negative encounters with providers may serve as a barrier to ongoing help-seeking, outweighing the potential benefits of treatment. This quote also reveals that prior negative experiences with providers may delay treatment engagement until acute care is absolutely needed. Thus, reinforcing a crisis-management mentality instead of prevention.

Another participant described an experience where a provider pushed medication to address symptoms without considering their perspectives or exploring alternative recovery pathways. The authoritative therapeutic approach left the participant feeling frustrated and disempowered, reinforcing skepticism around mental health providers: “*I’ve had the worst experience with psychiatry. Absolutely the worst experience because they wanted to push medication. And again, not that I have anything against that, but when someone does not want it, they should be respected as to why they do not want it*.” (Woman; Age 46; Not in treatment). This quote illustrates how noncollaborative/authoritarian approaches to treatment can lead to poor patient engagement, decreased *confianza*, and potentially worse mental health outcomes. Another participant similarly expressed discontent with the care they received, which they described as a failure in shared decision-making: “*A veces son [doctores] nuevos de la universidad y quieren que pruebe nuevos medicamentos. No soy un juguete para que me prueben medicamentos diferentes. Si ya saben que me estoy bebiendo un medicamento, déjenme como estoy. ¿Hacer una prueba conmigo? No, conmigo no haga pruebas, porque yo no soy un juguete; yo soy un humano*.” [*Sometimes there are new doctors from the university, and they want me to try a new medication. I am not a toy for them to be trying different medications. If they already know what medication I am taking, leave it as it is. Why try something new with me? Do not do that with me because I am not a toy. I am a human*.] (Man; Age 58; In treatment). This participant’s statement describes a therapeutic relationship that did not sufficiently elicit the patient’s preferences and concerns, leaving the patient feeling disrespected and thus highlighting the importance of person-centered, culturally responsive care.

##### 4.1.3.2 Lack of trust in providers and systems of care

Medical mistrust was a significantly discussed barrier to treatment engagement among most participants (*n =* 6 in treatment, *n =* 6 not in treatment), often shaped by negative encounters with providers and systems of care. Many participants reported difficulty being open or honest with providers for fear that their personal information would be used against them or shared without consent. One participant shared how the fear of losing custody of their children deterred them from being vulnerable with providers: “*I’ve been in abusive relationships, like domestic violence. Do you think I ever voiced that to a provider? Never, because my kids would have been taken. That was my fear. But the system does not make us feel trusted. I cannot tell you that I’ve ever been 100% honest with any therapist because there is no trust*.” (Woman; Age 46; Not in treatment). The participant’s words highlight an important Latino value and cornerstone of healing, *confianza* within the therapeutic relationship. This quote also underscores healthy cultural mistrust, a rational protective and survival coping response to historical and ongoing injustices endured by Latinos in the United States (Gallo et al., 2009).

For other participants, mistrust in providers and systems of care stemmed from negative messages about services, often communicated through trusted community sources. These messages often discouraged treatment-seeking behaviors and contributed to ambivalence around engaging with services. One participant described the narrative about providers shared within the community: “*Sí, muchas personas me dicen: ‘Ten cuidado con los psiquiatras porque los psiquiatras son como algunos médicos que te pueden hacer daño.’”* [*Yes, a lot of people have told me: “Be careful with psychiatrists because psychiatrists are like some doctors who can cause you harm.”*] (Woman; Age 66; In treatment). The participant’s statement illustrates the power of community messages, which may act as a significant barrier to seeking services. This excerpt also reveals how community messages can create a treatment gap, where individuals do not know where to turn for help or forgo care altogether.

##### 4.1.3.3 Mental health stigma

Across participants (*n =* 7 in treatment, *n =* 8 not in treatment), mental health stigma was another barrier often discussed within the context of culture, familial, and community dynamics. Participants described shame, judgment, and intergenerational transmission of mental health beliefs and attitudes, shaping perceptions of and engagement with services. One participant shared an oral message passed down from older generations about mental health treatment: “*Según los abuelos, los padres de antes decían que eso [los servicios de salud mental] es para los locos*.*”* [*According to my grandparents, priests from back then would say that mental health services are for crazy people*.] (Man; Age 58; Not in treatment). The participant’s comment highlights how mental health concerns have historically been framed as abnormal, leading families to echo the same message over time. The statement also reflects the Latino value of *respeto*, in which individuals tend to honor and follow the guidance of elders or those in positions of authority. Another participant emphasized how the lack of mental health knowledge contributed to mental health stigma: “*Qué no comprendemos también, porque vemos a una persona sufriendo de muchas cosas mentales y nosotros no las entendemos y las etiquetamos mal*.” [*We also do not understand, because sometimes we see a person suffering from mental conditions, and we do not understand them, and we label them poorly*.] (Woman; Age 60; In treatment). The participant’s words illustrate how the lack of understanding around mental health can lead individuals to misinterpret people’s lived experiences, contributing to harmful assumptions and stigma.

#### 4.1.4 Inner setting facilitators

##### 4.1.4.1 Mental health psychoeducation

Mental health psychoeducation was identified as a core facilitator of treatment engagement among most participants (*n =* 4 in treatment, *n =* 5 not in treatment). Many emphasized that receiving education on mental health symptoms, how to access services, confidentiality, and the therapy process would empower individuals to seek and utilize services. One participant explained how their perception of mental health treatment shifted from stigma-based to recognizing it as a valuable source of support after becoming more informed: “*Antes yo dije. “Ay, no, que eso es para locos.” Ahora, con la información que he oído en la televisión y en las noticias, de la gente que ha venido, como el psicólogo, ya tengo otro pensamiento, otro modo de pensar sobre cómo son las cosas: que si vas a un psicólogo no es porque estés loco. Es porque te dan un consejo. Mi manera de pensar cambió*.” [*Before, I would say. “That is for crazy people.” Now, with the information that I have heard from the television, news, and from the people who have presented here (psychologists), I have a different belief, another way of thinking about how things are: that if one goes to a psychologist, it is not because they are crazy. It is because they can give you advice. My way of thinking has changed*.] (Man; Age 58; Not in treatment). The participant’s comment illustrates how knowledge can improve attitudes toward mental health services. This quote also highlights the importance of receiving information from trusted sources who are viewed as credible and knowledgeable within the community.

Another participant shared that mental health outreach efforts focused on facilitating access to services and mental health in general could enhance treatment engagement: “*Yo creo, más que nada, hacer campañas, hacer campañas para que la comunidad entienda lo que es la salud mental. Campañas publicadas, campañas publicitarias*.” [*I think, more than anything, have campaigns, have campaigns so that the community understands what mental health is. Public campaigns, advertising campaigns*.] (Transwoman; Age 39; In treatment). The participant’s response reflects the importance of enhancing mental health and service literacy through broad public health campaigns. This excerpt also underscores the need to increase mental health awareness in the community to improve access to treatment.

##### 4.1.4.2 Providers who listen and are compassionate

All participants (*n =* 8 in treatment, *n =* 8 not in treatment) emphasized the importance of compassion and reflective listening from their providers. Participants described a need for providers to demonstrate care, deep empathy, and unconditional positive regard. For many, listening went beyond surface-level hearing; participants described a need for providers who engaged in deep, active, and empathic listening. One participant described how their providers’ deep investment in their recovery encouraged engagement with treatment: “*Si, ellos tomaron mucho interés en mi salud, de que yo me recuperara, de que no fuera a caer. Me tuvieron interés. Me motivaron.”* [Yes, they took a lot of interest in my health. So that I would recover and not relapse. They took an interest in me. They motivated me.] (Man; Age 68; Not in treatment). The participant’s statement highlights that when providers are fully invested in patient care, it can lead to improved outcomes, greater treatment adherence, and satisfaction. This quote also emphasizes the critical role of the therapeutic relationship in facilitating positive behavior change and treatment success.

Another participant underscored the value of providers who made ongoing efforts to better understand their lived experience: “*Yo puedo decir que mi terapeuta y psiquiatra, ellas siempre están como tratando de aprender más de mí. Siempre están tratando de escucharme y orientarme en el tratamiento*.” [I can say that my therapist and psychiatrist, they are always trying to learn more about me. They are always trying to listen to me and orient me to the treatment.] (Transwoman; Age 39; In treatment). The participant’s statement underscores the importance of attentive, person-centered care. The excerpt also highlights the ongoing relational process that fosters *confianza, respeto,* and collaboration within the patient-provider relationship.

#### 4.1.5 Individual characteristics facilitators

##### 4.1.5.1 Preference for behavior-based interventions

Preference for behavioral interventions emerged as an important facilitator of mental health treatment engagement across participants (*n =* 4 in treatment, *n =* 4 not in treatment) in both groups. Participants described a need and a preference for action-oriented interventions that incorporated body movement. One participant shared how body movement reduced stress and released built-up energy: “*Yo tengo que liberar energía y mantener mi mente ocupada para no pensar en cosas incorrectas. Por eso me gustaría que tuvieran talleres de baile para liberar energía*.” [*I must burn off energy and keep my mind busy so as not to think about things that are wrong. That is why I would like for there to be dance workshops, to burn off some energy.*] (Woman; Age 58; In treatment). The participant’s quote highlights the benefits of physical activity and the tangible improvements they deliver, which are valued more than abstract discussions. Another participant emphasized the value of engaging in pleasurable action-oriented activities to help with negative thoughts: “*Me ayuda todo el tiempo, porque cuando me siento down, cojo la sábana y empiezo a tejer y a tejer y ni me acuerdo de que voy a llorar, ni me acuerdo de que voy a gritar, ni me acuerdo de que voy a explotar porque mis emociones son diferentes*.” [*It helps me all the time, because when I feel down, I pick up the blanket and I start to sew, and I’ll sew, and I forget that I wanted to cry, I forget that I wanted to scream, I forget that I wanted to explode because my emotions are different*.] (Woman; Age 46; In treatment). The participant’s comment illustrates the important role of behavioral activation in emotion regulation. This excerpt also highlights the significance of practical, relatable strategies for managing psychological distress through concrete steps.

##### 4.1.5.2 Preference for faith-based care

All participants (*n =* 8 in treatment, *n =* 8 not in treatment) emphasized their preference for spiritually or religiously integrated treatment and its potential to enhance treatment engagement. Participants described the importance of working with providers who validated spiritual or religious beliefs and practices. One participant explained the benefits of working with providers who implemented a holistic approach to healing: “*And this is where holistic treatments are so different, because they offer an opportunity to speak with someone who incorporates spirituality without the limitation of the system*.” (Woman; Age 46; Not in treatment). The participant’s statement highlights the contrast between holistic approaches that integrate spirituality into healing and conventional methods that primarily focus on symptom reduction. This quote also underscores how traditional approaches to care may be restrictive or one-size-fits-all. Another participant similarly expressed the benefits of weaving together psychological tools and pharmacological medicine with spiritual resources: “*I feel that it could be beneficial to include prayer as part of my healing process, along with medication and the tools that a psychologist can provide*.” (Woman; Age 44; Not in treatment). The participant’s response illustrates how prayer can complement psychological and pharmacological interventions. The quote also highlights how prayer, alongside medical and psychological care, may enhance recovery pathways.

##### 4.1.5.3 Preference for context-based care

Preference for context-based care was also discussed as a facilitator of treatment engagement among participants (*n =* 4 in treatment, *n =* 7 not in treatment). Many emphasized the need for providers to tailor care to the current situation or the presenting problem. One participant articulated the need for providers to go beyond symptom management and develop a deeper understanding of how their illness affected daily life: “*This is not a knock at mental health providers, because I think that they do amazing work. But I think that the medical healthcare system is so book-focused. It’s focused on treating symptoms. And I feel they are just trying to remember what the text says about this condition and what the book says to tell people about their condition instead of getting to know them and addressing the real issue.”* (Woman; Age 46; Not in treatment). The participant’s statement emphasizes the importance of contextualized care, an approach that identifies and integrates a patient’s life context into treatment. This quote also highlights how focusing solely on symptoms can slow down the therapeutic working alliance by reducing *confianza*. Another participant similarly described the importance and need for providers to understand and consider the person’s context and lived experience: “*Lo que ellos (psicológicos) deberían saber es de dónde vienen, qué estado económico tienen, qué fue lo que vivieron, qué es lo que vinieron viviendo en su camino y qué es lo que están viviendo aquí*.” [*What they (psychologists) should know is where they come from, their socioeconomic status, what they lived, what they saw on their journey, and what they are living here.*] (Man; Age 29; Not in treatment). The participant’s statement reflects an important aspect of treatment engagement, the process of understanding a patient’s broader life context. This quote also highlights how focusing solely on symptoms may minimize the underlying social determinants of health influencing mental health concerns.

## 5 Discussion

The present study provides a detailed analysis of barriers to and facilitators of mental health treatment engagement among Latinos with SMI using the CFIR. Study participants identified two Inner setting barriers to treatment engagement: incongruence between treatment and Latino culture, and limited availability of Spanish-speaking and culturally responsive providers. Participants also identified an array of Individual characteristic barriers, including negative experiences with mental health providers, lack of trust in providers and systems of care, and mental health stigma, adding another layer of obstacles to treatment engagement. At the same time, several Inner setting facilitators to enhance treatment engagement were cited: mental health psychoeducation and providers who listen and are compassionate. Participants’ preference for behavior-based interventions, context-based care, and faith-based care further facilitated treatment engagement.

Several themes, including providers who listen and are compassionate, likely reflect universal preferences for high-quality mental health care. However, language, culture, stigma, and barriers to care shaped how Latino adults with SMI experienced and engaged in treatment. Consistent with the CFIR, our findings suggest that these determinants are not unique to this population but are influenced by the cultural and structural contexts in which treatment is provided.

While substantial research has focused on social determinants of health-related barriers to treatment engagement (Falgas et al., 2017; Green et al., 2020; Alegría et al., 2016; Cabassa et al., 2006), less attention has been paid to culturally incongruent care. Participants identified incongruence between treatment values and Latino cultural values as a key barrier to engagement, often noting that mainstream services would not meet their cultural needs. This belief was especially common among participants not in treatment. Prior research has shown that misalignment between Eurocentric frameworks and Latino cultural expectations undermines trust and rapport, limits treatment progress, and increases dropout rates (Interian et al., 2007; Kouyoumdjian et al., 2003; Organista, 2023; Vargas et al., 2015). Together, these findings suggest that cultural incongruence may undermine trust early in the help-seeking process, particularly among Latinos with SMI not yet engaged in care. They also extend prior work by demonstrating that cultural incongruence can deter engagement even prior to treatment initiation among Latinos with SMI. Clinically, these findings underscore the importance of cultural responsiveness in early service encounters to foster trust and improve engagement among Latinos with SMI.

The limited availability of Spanish-speaking providers is a well-documented barrier to engagement in mental health treatment among Latinos (Cabassa et al., 2006; Alegría et al., 2002; Sentell et al., 2007; Stafford and Draucker, 2020). Our findings align with prior work suggesting that the mental health workforce is not well-positioned to meet the demand for culturally responsive, multilingual care (Gustafson et al., 2025). Although interpreter services may help address gaps in the availability of bilingual providers (Fennig and Denov, 2021; Moreno and Morales, 2010; Taknint et al., 2023), participants raised concerns about interpreter quality, including proficiency and competence. Together, the scarcity of culturally responsive providers and concerns about interpreter quality may contribute to perceptions that care is unavailable to Latinos with SMI. This may delay treatment engagement, prolong recovery, and shift the burden of illness management onto individuals. Although bilingual providers are essential, linguistic concordance alone is insufficient without culturally responsive care and ongoing commitment to cultural humility. Clinically, our findings highlight the need to expand and diversify pathways into mental health careers for bilingual individuals to meet growing workforce demands. This includes building early interest through undergraduate outreach, mentorship, and experiential opportunities that cultivate sustained interest in culturally responsive care.

Beyond structural barriers and stigma (Alegría et al., 2016; Interian et al., 2010), negative experience with providers and lack of trust in providers and systems of care were cited as barriers to treatment engagement. Consistent with our findings, other Latino populations have identified negative experiences with treatment, providers, and therapy as barriers to treatment initiation and adherence (Stafford and Draucker, 2020; Newberry et al., 2024). Research has also shown that such experiences can undermine trust, weaken the therapeutic alliance, and reduce perceptions of provider trustworthiness (Kouyoumdjian et al., 2003; Newberry et al., 2024). Together, these findings suggest that negative experience with mental health providers and mistrust may delay future help-seeking and reduce confidence in provider recommendations. Importantly, these findings highlight that trust-building is an ongoing process that merits clear delineation as a treatment objective in the overall treatment plan and initial contact (Añez et al., 2008). These findings refine prior research by showing how negative provider encounters and culturally grounded mistrust operate relationally to shape treatment engagement and confidence in care. Clinically, this underscores the need to rebuild trust between Latino communities and systems of care. This can be achieved through intentional community engagement efforts and investment in provider training that emphasize person-centered care and *personalismo*.

The findings of our study further support previous research on facilitators of treatment engagement among Latinos with SMI (Corrigan et al., 2017; Corrigan et al., 2018; Vargas et al., 2015; Cabassa et al., 2019; López et al., 2009). Our participants described the importance of receiving psychoeducation on the therapeutic process, treatment access, and symptom recognition as a strategy to improve treatment engagement. Participants also emphasized outreach campaigns as a method for disseminating mental health psychoeducation. Prior research with Latinos has shown a positive association between mental health psychoeducation and improved mental health outcomes (López et al., 2009; Pérez-Flores and Cabassa, 2021; Sanchez et al., 2021). Research has also found mental health psychoeducation to improve knowledge of symptoms (López et al., 2009) and decrease stigma (López et al., 2009; Cabassa et al., 2015) among Latinos. Together, these findings suggest that mental health psychoeducation may enhance treatment initiation among Latinos with SMI. Importantly, these findings highlight the need for culturally responsive psychoeducational material that goes beyond brochures or informational sheets focused on enhancing mental health literacy. These findings add to prior research by suggesting that psychoeducational materials for Latinos should guide individuals through each stage of treatment, from symptom recognition to transition out of care. Clinically, these findings highlight the need for mental health clinics to strengthen their presence in Latino communities through health fairs, workshops, and tailored campaigns.

As with many other studies (Uebelacker et al., 2012; Stafford and Draucker, 2020), our participants reported on the importance of working with caring mental health providers. Participants underscored that access to empathetic providers who practice reflective listening facilitates both treatment initiation and long-term engagement in care. Prior research has shown that Latinos who perceive providers as caring, compassionate, and action-oriented are more likely to experience positive treatment outcomes (Organista, 2023; Alegría et al., 2014; Cortes et al., 2009). Additional research has also found that Latinos are more likely to adhere to treatment when providers foster collaboration and actively engage them in shared decision-making (Hale et al., 2020). Together, these findings suggest that access to providers who are collaborative, compassionate, accepting, and empowering (Miller and Rollnick, 2023) is key to facilitating treatment initiation and sustaining engagement in care. These findings deepen prior work by showing that provider characteristics actively shape treatment engagement, influencing initiation, therapeutic relationships, and adherence. Clinically, these findings highlight the importance of training and supporting providers to deliver care through a person-centered lens.

Study findings extend the literature by showing that active, spiritually meaningful, and context-based approaches are key facilitators of treatment engagement. For participants, context-based care meant centering treatment on the concerns they identified and sought to address, rather than primarily on provider-defined symptoms. Consistent with our findings, prior research shows that Latinos prefer action-oriented and spiritually integrated interventions and care grounded in self-identified concerns (Vargas et al., 2015; Benson-Flórez et al., 2017; Turner and Llamas, 2017; Vargas et al., 2019). These findings suggest that Latinos are likely to engage in care that is active, spiritually integrated, and aligned with their own priorities (Vargas et al., 2015; Vargas et al., 2019). Findings also suggest that even Latinos who did not identify with specific religious or spiritual traditions preferred treatment that incorporated a religious or spiritual component. This can be partially attributed to the interplay among familial ties, cultural identity, cultural syncretism, and community cohesion. Another possible consideration is the salience of Latino cultural values (e.g., *familismo*) as the context for religious beliefs, which may function independently of religious structures (Campesino and Schwartz, 2006). This work adds to prior research by demonstrating that engagement is strengthened when care is not only culturally and spiritually responsive but also explicitly behavioral-focused and grounded in patients’ goals (Alegría et al., 2014; Benson-Flórez et al., 2017). Together, these findings suggest that engagement among Latinos with SMI may be driven less by structural access alone and more by alignment between care, lived context, and relational trust within treatment encounters. Clinically, this suggests that engagement improves when care is aligned with how patients define their problems and preferred paths to healing.

### 5.1 Strengths and limitations

The present study has several strengths and limitations worth noting. First, to our knowledge, this is the first qualitative study to explore barriers to and facilitators of treatment engagement among Latinos with SMI using the CFIR. Second, unlike most depression-focused studies, our sample included Latinos with varied SMI, broadening the understanding of treatment engagement across psychiatric conditions. Third, our sample included both monolingual Spanish-speaking and bilingual Latinos with SMI. Fourth, our sample included an even number of participants in treatment and not in treatment. Fifth, our findings go beyond commonly documented social determinants of health (SDOH)-related barriers by highlighting the relational and intervention-level factors that shape treatment engagement.

Despite these strengths, the present study has several limitations. First, although the CFIR helps identify barriers and facilitators, it cannot determine which factors most strongly influence treatment engagement or how they interact. Second, our findings were also limited to two CFIR domains, leaving barriers and facilitators within the Outer setting, Innovation, and Process domains unexplored. This may, in part, be attributed to participants’ limited knowledge and understanding of the macro-level (Outer setting) factors (e.g., policies, finances, sociocultural environment) that influence service implementation efforts. Similarly, participants are shielded from the intricacies (e.g., planning, execution, methodology) of the implementation process (Innovation and Process), making it difficult for them to address the overarching rollout of services. To address untapped CFIR domains, future studies may consider interviewing organizational leadership, providers, and staff. Third, our sample had an uneven proportion of women and men, thus preventing gender comparisons of barriers to and facilitators of treatment engagement. In addition, participants in treatment and not in treatment differed in diagnostic composition. Differences in diagnostic composition may have influenced participants’ experiences and treatment engagement, making it difficult to disentangle the effects of diagnosis from those of treatment status. However, diagnostic differences alone are unlikely to explain the findings, as referral source and social factors may also have influenced lived experience and treatment engagement. Future studies with more diagnostically comparable groups are needed to better disentangle these influences. Fourth, the use of single interviews limited insight into how treatment barriers and facilitators changed over time and across lived experiences. Fifth, the study captures only participant perspectives and does not include provider, family, or system-level viewpoints, which may shape engagement differently. Sixth, the uneven representation of Latino subgroups across study groups may limit the interpretation of the findings. The not-in-treatment group was predominantly composed of participants from Puerto Rico, whereas the treatment-engaged group included participants from multiple Latino backgrounds. Differences in migration histories, citizenship experiences, sociocultural contexts, and access to resources may have influenced treatment engagement and the themes identified, limiting the generalizability of findings to Latino individuals with SMI more broadly. Seventh, we did not obtain a referral source (self-referral, referral, or mandate) for participants in treatment, which may affect the engagement process.

## 6 Conclusion

This study advances the literature by applying an implementation science framework to understand treatment engagement among Latinos with SMI, focusing on relational and intervention-level mechanisms often overlooked in prior research. These findings contribute to existing literature by shedding light on non-SDOH-related barriers to and facilitators of treatment engagement for Latinos with SMI. Findings emphasize the importance of minimizing barriers and maximizing facilitators that fall directly within the purview of the provider and clinic.

## Data Availability

The datasets for this manuscript are not publicly available due to confidentiality regarding the involvement of a vulnerable population. Requests to access the datasets should be directed to Oscar F. Rojas Perez, oscar.rojasperez@yale.edu.
