# Supplementary material for: Confianza, culture, and care: barriers to and facilitators of mental health treatment engagement among Latinos with serious mental illness
Source: Front Psychol. 2026 Aug 21;17:1904976. doi: 10.3389/fpsyg.2026.1904976 (PMC13541748; doi:10.3389/fpsyg.2026.1904976)
Supplement: Supplementary file 1 [file Supplementary_file_1.pdf]

Appendix A

Abriendo Caminos: Qualitative Interview Questions
(Treatment and No Treatment)

Disclaimer
For the purpose of the study, we use the term Hispanic or Latino when referring to individuals with Latin American origins. However, we also understand that not everyone with Latin American heritage identifies with Hispanic or Latino. In an effort to be culturally responsive and gender inclusive throughout this interview, please let us know how you identify (e.g., Hispanic, Latina, Latine, Latino, Latinx).

Definitions
a. Latina/o/e/x: People or community with Latin American origins or descent
b. Serious mental illness (SMI): cluster of disorders (e.g., schizophrenia, bipolar disorder, and recurrent major depression)
c. Formal mental health treatment: Treatment with a licensed provider and/or medication
d. Cultural forms of treatment: These include prayer, self-reliant attitudes, herbal medicine, etc
e. Peers: Individuals with lived experience of mental health (e.g., SMI) and mental health treatment who provide services (coaching, navigation of services, group facilitation, etc.)
f. Latino cultural values:familismo, respeto, personalismo, confianza, simpatía, etc.

Prompts for following up with a participant's response or probing
a. Please give an example
b. Please provide another example
c. What does that look like
d. In what way
e. What else
f. Would else would you like to add
g. Please elaborate more
h. What are the benefits
i. What are some challenges

Patient needs
1. When you think of mental health, what are the first things that come to mind?
2. How well prepared do you think mental health providers/clinics are to address the mental health needs of Latinos with SMI and trauma?
3. What changes or alterations are needed in mental health service(s)/treatment(s) to engage Latinos with SMI and trauma in mental health services?

    3.1. What should mental health treatment look like for Latinos with SMI and trauma?
    3.2. What should mental health providers be aware of when working with Latinos with SMI and trauma?

Treatment characteristics
4. How do people in your community/family/society talk about mental health treatment?

    4.1. How do these messages influence your decision to engage in treatment?

5. What are the advantages of formal mental health treatment?

    5.1. What are the disadvantages of formal mental health treatment?

6. What are the advantages of cultural forms (e.g., prayer, going to the priest, spiritual dances) of mental health treatment?

    6.1. What are the disadvantages of cultural forms (e.g., prayer, going to the priest, spiritual dances) of mental health treatment?

Treatment compatibility

7. What Latino values are most important to you? *If the participant is unable to come up with a value provide examples* (*familismo, respeto, personalismo, confianza, simpatía*).
8. How do current mental health treatment(s)/service(s) fit with Latinos values (insert values mentioned above) or practices?

    8.1. What barriers to mental health treatment might Latino cultural values and/or practices create?

9. What do you think are relevant mental health needs in the Latino community?

    9.1. How can mental health treatment(s)/service(s) be improved to better engage and address the mental health (insert needs mentioned above) of Latino communities?

10. How confident are you with the capability of current mental health treatment(s)/service(s) to address the mental health needs of Latinos with SMI and trauma?
11. What are the advantages of receiving guidance/help from a Latino peer with lived experience of SMI and trauma?
12. What are the disadvantages of receiving guidance/help from a Latino peer with lived experience of SMI and trauma?
13. How would you feel receiving guidance/help from a Latino peer with lived experience of SMI and trauma?
